# The Immunology of Macrophage Activation Syndrome

**DOI:** 10.3389/fimmu.2019.00119

**Published:** 2019-02-01

**Authors:** Courtney B. Crayne, Sabrin Albeituni, Kim E. Nichols, Randy Q. Cron

**Affiliations:** ^1^Pediatric Rheumatology, University of Alabama Birmingham, Birmingham, AL, United States; ^2^Department of Oncology, St. Jude Children's Research Hospital, Memphis, TN, United States

**Keywords:** macrophage activation syndrome, hemophagocytic lymphohistiocytosis, cytokine storm, IL-1, IL-6, IL-18, NK cell, anakinra

## Abstract

Synonymous with secondary hemophagocytic lymphohistiocytosis, macrophage activation syndrome (MAS) is a term used by rheumatologists to describe a potentially life-threatening complication of systemic inflammatory disorders, most commonly systemic juvenile idiopathic arthritis (sJIA) and systemic lupus erythematosus (SLE). Clinical and laboratory features of MAS include sustained fever, hyperferritinemia, pancytopenia, fibrinolytic coagulopathy, and liver dysfunction. Soluble interleukin-2 receptor alpha chain (sCD25) and sCD163 may be elevated, and histopathology often reveals characteristic increased hemophagocytic activity in the bone marrow (and other tissues), with positive CD163 (histiocyte) staining. A common hypothesis as to the pathophysiology of many cases of MAS proposes a defect in lymphocyte cytolytic activity. Specific heterozygous gene mutations in familial HLH-associated cytolytic pathway genes (e.g., *PRF1, UNC13D*) have been linked to a substantial subset of MAS patients. In addition, the pro-inflammatory cytokine environment, particularly IL-6, has been shown to decrease NK cell cytolytic function. The inability of NK cells and cytolytic CD8 T cells to lyse infected and otherwise activated antigen presenting cells results in prolonged cell-to-cell (innate and adaptive immune cells) interactions and amplification of a pro-inflammatory cytokine cascade. The cytokine storm results in activation of macrophages, causing hemophagocytosis, as well as contributing to multi-organ dysfunction. In addition to macrophages, dendritic cells likely play a critical role in antigen presentation to cytolytic lymphocytes, as well as contributing to cytokine expression. Several cytokines, including tumor necrosis factor, interferon-gamma, and numerous interleukins (i.e., IL-1, IL-6, IL-18, IL-33), have been implicated in the cytokine cascade. In addition to broadly immunosuppressive therapies, novel cytokine targeted treatments are being explored to dampen the overly active immune response that is responsible for much of the pathology seen in MAS.

## Introduction

Synonymous with secondary hemophagocytic lymphohistiocytosis (HLH), macrophage activation syndrome (MAS) is a term used by rheumatologists to describe a potentially life-threatening complication of systemic inflammatory disorders, most commonly systemic juvenile idiopathic arthritis (sJIA) and its adult equivalent, adult onset Still disease. This syndrome was first reported in juvenile rheumatoid arthritis (JRA) patients [now termed juvenile idiopathic arthritis (JIA)] with enlarged Kupffer cells (i.e., stellate macrophages in the liver) who concomitantly suffered from strikingly low counts of white blood cells and unusually low erythrocyte sedimentation rates (ESR) (1). Subsequent literature described the presence of activated macrophages and hemophagocytic histiocytes in patients with rheumatic disease, termed reactive hemophagocytic syndrome and now known as MAS (2–4).

A majority of clinical data available involves MAS as a complication of sJIA. The prevalence of fulminant MAS in patients with sJIA is reported to be about 10%; however, subclinical MAS may be present in as many as 30% of children with known or suspected sJIA (5–8). As MAS becomes more clinically recognized, an increasing frequency of occurrence in other systemic inflammatory disorders [i.e., systemic lupus erythematosus (SLE), Kawasaki disease, and periodic fever syndromes] has been reported (9–11). While MAS is known to complicate a variety of inflammatory conditions, including but not limited to malignancy, infection (i.e., Epstein-Barr virus), and primary immunodeficiencies, it is most commonly reported as a well-recognized complication of sJIA, and therefore, much of the understanding of the genetics, pathology, and subsequently immunology is derived from this specific cohort (12).

Early recognition of MAS remains diagnostically challenging as there is no diagnostic test or even a set of disease uniform diagnostic criteria to differentiate MAS from the underlying systemic inflammatory condition. Clinical and laboratory features of MAS include sustained fever, hyperferritinemia, pancytopenia, fibrinolytic consumptive coagulopathy, and liver dysfunction. In 2016, an expert consensus panel published a set of validated diagnostic criteria to help distinguish a sJIA flare from MAS. The final MAS criteria for children with sJIA proved to be both sensitive (0.73) and specific (0.99). The diagnosis of MAS can be made in a febrile patient with sJIA, or suspected sJIA, who has a serum ferritin level > 684 ng/ml plus any 2 of the following: platelet count ≤ 181 × 109/l, aspartate aminotransferase > 48 units/l, triglyceride concentration > 156 mg/dl, or fibrinogen ≤ 360 mg/dl (5, 6). These relatively few total criteria are routinely readily available and timely. To date, these criteria have yet to prove diagnostic in other autoimmune diseases and remain limited to children with known or suspected sJIA, with the possible exception of adult onset Still disease (13).

The clinical similarity of MAS and secondary HLH has led some clinicians to use the longer-standing HLH-2004 diagnostic guidelines, which require five of the following eight criteria to be met for diagnosis: fever, splenomegaly, cytopenias (affecting ≥ 2 of 3: hemoglobin < 90 g/l, platelets < 100 × 109/l, neutrophils < 1.0 × 109/l), hypertriglyceridemia (≥265 mg/dl) and/or hypofibrinogenemia (≤ 1.5 g/l), hemophagocytosis in bone marrow or spleen or lymph nodes, low or absent natural killer (NK) cell activity, ferritin ≥ 500 μg/l, and sCD25 ≥ 2,400 units/ml (14). Using this strict set of criteria may delay diagnosis in patients with a less severe initial presentation.

**Figure 1:**
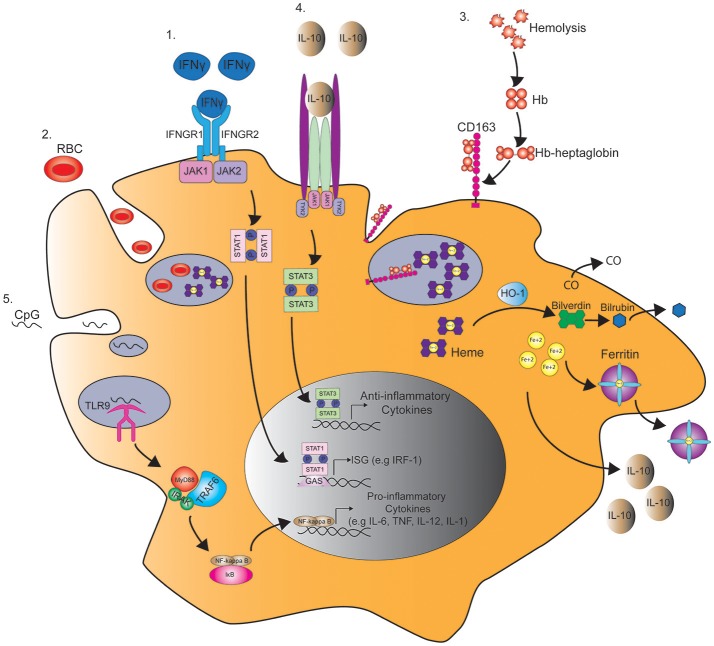
Pathways regulating macrophage function in MAS. 1. IFNγ binds the IFNγ receptor (IFNGR) and subsequently induces the phosphorylation of STAT1 by JAK1/2 in the cytoplasm. STAT1 dimer then binds to γ-interferon activation site (GAS) and enhances the transcription of interferon-stimulated genes (ISG), such as interferon regulatory factor 1 (IRF1). 2. STAT1 activation by IFNγ also induces macropinocytosis leading to the engulfment and degradation of red blood cells (RBC) in a process known as hemophagocytosis. 3. Hemophagocytosis is also mediated by the uptake of hemoglobin (Hb)-heptaglobin complex by CD163. The Hb-heptaglobin complex is degraded in the lysosome followed by catalysis of heme by heme oxygenase-1 (HO-1) to carbon dioxide (CO), bilverdin, and iron (Fe^2+^). Bilverdin is then converted to bilirubin by bilverdin reductase, and iron is bound to ferritin. 4. This process also leads to the production of IL-10 that through binding to IL-10 receptor induces STAT3 phosphorylation and the production of anti-inflammatory cytokines that counteract IFNγ signaling. 5. In a mouse model of MAS, serial injections of CpG induce the activation of toll-like receptor 9 (TLR9) in the macrophage endosome leading to the production of pro-inflammatory cytokines in a MyD88 and NFκB dependent manner.

Hemophagocytosis is defined as the engulfment of blood cells, including red blood cells (RBC), white blood cells, or platelets by phagocytic cells (Figure 1). Hemophagocytosis by macrophages has been widely associated with the development of MAS in patients with sJIA and other rheumatologic diseases (15–17). Histopathology often reveals characteristic increased hemophagocytic activity in the bone marrow, liver, and spleen with positive CD163 (histiocyte) staining, although hemophagocytosis may not be present in initial stages and is neither sensitive nor specific for MAS (18–20). Detection of hemophagocytosis using serum laboratory tests includes soluble interleukin 2 receptor alpha chain (sCD25) and soluble CD163 (sCD163), a high affinity scavenger receptor for hemoglobin-haptoglobin complexes (Figure 1), both of which may be elevated, thereby suggesting sCD25 and sCD163 to be more sensitive in detection of MAS. These tests are only performed at select sites, making them costly with a long turnaround time for results thus leading to a delay in diagnosis and ultimately treatment (18). If inadequately treated, MAS can result in multi-organ failure and death. In the absence of universal diagnostic criteria or a gold-standard laboratory test, understanding the immune mechanisms of MAS may lead to more prompt recognition and target-specific therapies.

## Cytolytic Cell Dysfunction in MAS

MAS shares many etiologic similarities with familial HLH (fHLH), also referred to as primary HLH, not the least of which is the increased prevalence of heterozygous mutations in known fHLH genes that are now being increasingly recognized in MAS patients. fHLH is a severe form of cytokine storm syndrome occurring in infancy, typically within the first few days to months of life. fHLH is a result of homozygous, or compound heterozygous, mutations in genes involved in the perforin-mediated pathway of cytolysis shared by NK cells (innate immunity) and cytotoxic CD8 T cells (adaptive immunity) (21).

The first gene recognized to contribute to fHLH was *PRF1* which gives rise to perforin (22). Homozygous defects in *PRF1* were identified in several families with fHLH (23). Normally, perforin is packaged into cytolytic granules and upon NK cell or CD8 T cell activation is trafficked along the actin cytoskeleton to the immunologic synapse between the cytolytic lymphocyte and the antigen presenting cell (APC) or target cell (24). A variety of fHLH genes are involved in trafficking and docking of the cytolytic granules, including *LYST, RAB27A, UNC13D, STXBP2, STX11*, and others, to the cell membrane (Table 1). The polarized granules then allow release of perforin into the synapse to form a pore between the lytic cell and the target cell. Granzyme B, which is co-packaged with perforin, is then delivered to the target cell, resulting in apoptotic cell death. Homozygous disruption of any of the critical genes involved in this process of perforin-mediated cytolysis (Table 1) results in fHLH, occurring in about 1 in 50,000 live births and often associated with an infectious trigger. The inability to lyse the infected APC results in a prolonged interaction between the cytolytic lymphocyte and the APC yielding a pro-inflammatory cytokine storm believed to be responsible for the clinical features of fHLH (31). Human fHLH has been modeled in *PRF1* deficient mice infected with LCMV, and both CD8 T cells and interferon-gamma (IFNγ), a cytokine known to be the main driver of anemia in models of fHLH and fulminant MAS (32, 33), were found to be critically important mediators of mouse mortality (34). IFNγ and its downstream JAK pathways are both considered as possible targets for therapy in man (Table 2). IL-33, a member of the IL-1 family of cytokines, may also play a role in T cell hyperactivation during HLH (Table 2) (42).

**Table 1:** Cytolytic pathway genes associated with HLH and MAS.

**Gene**	**Protein**	**Function**
*PRF1*	Perforin	Pore formation (23)
*UNC13D*	Munc13-4	Vesicle priming (25)
*STX11*	Syntaxin 11	Vesicle docking (26)
*STXBP2*	Munc18-2	Vesicle membrane fusing (27)
*LYST*	Lysosomal trafficking regulator	Vesicle sorting (28)
*RAB27A*	Rab27a	Vesicle fusing (29)
*AP3B1*	AP-3	Vesicle trafficking (30)

**Table 2:** MAS therapies directed at cytokine blockade and disruption of cell–cell interactions.

**Reported cytokine target**	**Therapeutic mechanism**	**Example**
IL-1	IL-1 receptor antagonist	Anakinra, canakinumab (35)
IL-6	Anti-IL-6R monoclonal Ab	Tocilizumab (36, 37)
IL-18	IL-18 binding protein	Not commercially available (38)
CD28	CTLA4-Ig	Abatacept (39)
JAK1/2	JAK inhibitor	Tofacitinib (40)
**Theoretical cytokine target**	**Proposed mechanism**	**Example**
IL-10	Recombinant IL-10 protein	None available (41)
IL-33	Anti-IL-33R monoclonal Ab	None available (42)
IFNγ	Anti-IFNγ monoclonal Ab	None available (34, 43)

*TNF, tumor necrosis factor; Ab, antibody; IL, interleukin; R, receptor; CTLA, cytotoxic T-lymphocyte-associated protein 4; Ig, immunoglobulin; JAK, Janus kinase; IFNγ, interferon-gamma*.

MAS or secondary HLH is much more common than fHLH and occurs in children and adults (44). Interestingly, heterozygous mutations in fHLH genes may be found in upwards of 40% of individuals with secondary HLH and MAS (45, 46). Some of these mutations are hypomorphic in nature, even those identified in genetic regulatory regions (47, 48), and others have dominant-negative effects (49, 50). Like in fHLH, these heterozygous gene mutations alter cytolytic function in NK cells, and presumably CD8 T cells as well. A combination of a chronic inflammatory state, such as in sJIA or SLE, with a genetic predisposition, and/or a triggering infection may result in fatal MAS or sHLH. Examples of this include identification of heterozygous fHLH gene mutations in patients with fatal influenza (H1N1) infections and associated hemophagocytosis (51), and increased percentages of *PRF1* and *UNC13D* heterozygous mutations in cohorts of sJIA patients who develop MAS (52, 53). This has led investigators to propose a threshold model of MAS, in which combinations of genetic predisposition, an underlying inflammatory state, and triggering infectious agents, results in a clinically relevant cytokine storm syndrome (54). Thus, genetic defects in cytolytic lymphocytes of the innate (NK cells) and adaptive (CD8 T cells) immune system can contribute to MAS. Moreover, there are other mechanisms by which MAS can be triggered by genetic mutations that directly affect cells (e.g., macrophages and dendritic cells) of the innate immune system through altering cytokine production via the inflammasome complex (55).

## Macrophages in MAS

As the name implies, macrophage activation is a definitive characteristic of MAS (Figure 1). The role of macrophages in MAS has been largely established through their mediation of hemophagocytosis and hypercytokinemia. However, their potential role in dampening an overly exuberant immune response has also been suggested (56).

### Hemophagocytosis

Despite the reported increase in hemophagocytic macrophages in the bone marrow and liver of sJIA and MAS patients, there are conflicting reports on the role of hemophagocytic macrophages in disease pathology induction. Several studies have shown that hemophagocytic macrophages induce pathogenesis. The cause of red blood cell (RBC) destruction in hemophagocytic syndromes is largely attributed to activated macrophages. In a model of autoimmune hemolytic anemia, treatment with liposomal chlodronate increased RBC counts by blocking the ability of macrophages to phagocytose RBC (57). Interestingly, hemophagocytosis was induced in macrophages treated with IFNγ (58). In addition, hemophagocytosis did not develop in two HLH patients with IFNγ receptor deficiency (59). Hemophagocytic macrophages were also found to produce the pro-inflammatory cytokine tumor necrosis factor (TNF) in the liver biopsy of MAS patients (60). Since both IFNγ and TNF are key cytokines for the polarization of classically activated or pro-inflammatory M1 macrophages (61, 62), these findings suggest that hemophagocytic macrophages in MAS could have an M1 phenotype.

The identification of hemophagocytic macrophages in bone-marrow aspirates and liver biopsies of MAS patients largely relies on histochemical analysis of CD163 staining. CD163 is an exclusive marker of cells of the monocyte/macrophage lineage. It is often expressed in activated macrophages but is not restricted to hemophagocytic macrophages (63). As previously mentioned, CD163 is a hemoglobin scavenger receptor that mediates the endocytosis of haptoglobin-hemoglobin complexes (64). Avcin et al. reported the increased frequency of CD163^+^ hemophagocytic macrophages in three MAS patients who developed SLE, sJIA, and Kawasaki disease (65), suggesting that CD163 could be a diagnostic marker in MAS. In contrast, Behrens et al. demonstrated that CD163 expression was increased in the bone-marrow aspirates of 15 sJIA patients, of which two patients were diagnosed clinically with overt MAS, thereby suggesting that this increase is not exclusive to MAS patients. Interestingly, activated or hemophagocytic CD163^+^ macrophages within the bone-marrow aspirates preceded the development of full-blown MAS, thus supporting the hypothesis that occult MAS could precede clinical MAS in sJIA patients (8). These findings further suggest that MAS and sJIA disease flare may be two ends of the same spectrum with MAS at the most extreme (66).

Since CD163 expression is increased during active sJIA, the ability of activated macrophages to shed this protein (67, 68) led to further speculations on the use of soluble CD163 (sCD163) as a diagnostic marker of macrophage activation. Several studies have reported that sCD163 is increased in the serum of sJIA patients and correlates with an increase in sCD25 and ferritin and with low platelet counts at disease peak (18, 69). Sakumura et al. reported increased levels of serum sCD163 in patients diagnosed with confirmed sJIA and MAS compared to patients with acute sJIA in the absence of MAS, suggesting a correlation between sCD163 levels and clinical MAS (70). Serum sCD163 shows promise as a diagnostic biomarker for MAS, although additional studies are needed to determine clinical significance.

In contrast to these findings, other studies have suggested that hemophagocytic macrophages have an M2 phenotype. Infusion of the M2-driving cytokine IL-4 with a micro-pump induced hemophagocytosis by macrophages. Surprisingly, hemophagocytosis was not inhibited by IFNγ blockade, and macrophages in IL-4 infused mice expressed arginase-1, a classical marker of M2 macrophages (71). Similarly, other reports have shown that CD163^+^ macrophages have anti-inflammatory M2 properties. The anti-inflammatory cytokine IL-10 was found to upregulate the expression of CD163 expression on macrophages (72). In addition, CD163^+^ macrophages are thought to play a protective role during inflammation due to their ability to clear free-hemoglobin (Hgb). Free Hgb binds to haptoglobin, which is then engulfed by macrophages through CD163-mediated endocytosis. This subsequently leads to the production of the anti-inflammatory agents, interleukin-10 (IL-10) and heme oxygenase (HO-1), by macrophages (73, 74) (Figure 1). HO-1 may also have anti-inflammatory effects by mediating the catabolism of heme to carbon monoxide (CO) and free iron (Fe^2+^) (Figure 1). Interestingly, in *in vitro* studies macrophages exposed to CO prior to lipopolysaccharide (LPS) stimulation have enhanced production of IL-10 and inhibited production of TNF (75) (Figure 1).

Similarly, ferritin is also considered to be cytoprotective through its ability to sequester free Fe^2+^, therefore decreasing endothelial apoptosis mediated by increased oxidative stress (76) (Figure 1). Moreover, studies in animal models of MAS favor the anti-inflammatory role of IL-10, since blockade of IL-10 in mice treated with serial injections of CpG worsen disease and induce symptoms of fulminant MAS (33, 41). Overall, these findings suggest that the increased numbers of CD163^+^ hemophagocytic macrophages and ferritin in MAS may be a compensatory mechanism rather than a cause of disease pathology in MAS.

In summary, the macrophage phenotype resides along a spectrum, due in part to the plasticity of macrophages. There are constant functional changes that occur in macrophages in response to changing stimuli during the progression of inflammation (77). In this line, the pro-inflammatory M1 and anti-inflammatory M2 phenotypes are considered to be two extremes of a continuous spectrum of various phenotypes that are finely tuned in response to external stimuli (78, 79). The degree of macrophage activation in MAS may therefore be reflective of the heterogeneity of macrophages within the inflammatory environment. Hemophagocytosis occurs in later stages of MAS and is only found in about 60% of HLH and MAS patient biopsies (80). This suggests that as the disease progresses, macrophages may switch from a pro-inflammatory to an anti-inflammatory phenotype, thereby balancing the extremely hyperactive inflammatory environment in patients with fulminant disease. Further investigations are needed to determine the role of hemophagocytic macrophages in the setting of MAS.

### Hypercytokinemia

The acute phase of MAS is often associated with markedly elevated levels of pro-inflammatory cytokines. This cytokine storm triggers a cascade of inflammatory pathways that, if untreated, leads to tissue damage and death (81). The working hypothesis suggests macrophages/monocytes produce a cocktail of cytokines, notably TNF and various interleukins (i.e., IL-6, IL-1β, and IL-18), which triggers a cascade of inflammatory pathways and ultimately creating a cytokine storm (Figure 1). TNF is a pro-inflammatory cytokine that drives macrophage polarization toward the M1-end of the spectrum. This cytokine has been described as being an anti-M2 factor due to its ability to inhibit STAT6-dependent M2 gene expression in tumor models (62, 82), therefore, favoring macrophage polarization of the M1 phenotype. Macrophages are also thought to be the main source of TNF in MAS.

*In situ* expression of TNF by hemophagocytic macrophages was reported in the liver of MAS patients (60). Elevated levels of TNF have been found in patients with other rheumatic diseases [i.e., rheumatoid arthritis (RA)], making it a prime target for treatment. Anti-TNF biologics are a class of medications that target TNF directly as monoclonal antibodies or the TNF receptor to block the cytokine cascade and successfully modify disease activity in a milieu of rheumatic diseases (e.g., RA, JIA, uveitis) (83, 84). While successful treatment of MAS with etanercept, a TNF receptor antagonist, has been reported (85) (Table 2) other studies have shown that it may trigger or worsen disease progression (86, 87). Thus, the role of TNF and its blockade in MAS remains unclear.

Like TNF, IL-6 producing macrophages have been found in the liver of MAS patients (60). Increased levels of IL-6 have also been reported in the serum of sJIA and in sepsis patients (88–90). Despite the association of IL-6 levels and MAS, the role of IL-6 in the pathogenesis of disease is not well-understood. It remains unknown whether macrophages are the main cellular sources of IL-6 in MAS patients. A recent study by Norelli et al. demonstrated that human monocytes are the primary producers of IL-1β and IL-6 in cytokine release syndrome and that ablation of monocytes could be protective (91) (Table 2). In contrast, IL-6 in combination with GM-CSF drives the differentiation of suppressive monocytic myeloid-derived suppressor cells (M-MDSC) in bone marrow (92).

Tocilizumab is a monoclonal antibody targeting the IL-6 receptor and is approved for use in RA, giant cell arteritis, polyarticular JIA, and sJIA (93). Despite its success in treating acute sJIA, patients with sJIA treated with tocilizumab remain at risk for MAS, arguing that IL-6 blockade alone is insufficient to control the inflammatory cascade (36, 94, 95). These patients tended to be afebrile and had lower cell counts and ferritin levels with higher liver enzymes (94, 96). The mechanism of IL-6 in the pathogenesis of MAS remains controversial. IL-6 likely contributes to the cytokine storm, but its role in clinical disease manifestations of MAS is limited, thus making it a questionable target for therapy.

As members of the IL-1 family of cytokines, IL-1β and IL-18 are potent inducers of IL-6 production in monocytes and macrophages (97, 98). Levels of IL-1β and IL-18 are markedly increased in patients with active sJIA and MAS (99–103). Anakinra is a recombinant IL-1 receptor antagonist used off-label in patients with sJIA and less commonly in patients with MAS, either in combination with sJIA or secondary other etiology (35, 104, 105). Efficacy data in the treatment of MAS with anakinra is limited to case reports and series, but many patients achieve disease remission with normalization of lab abnormalities and fever despite prior poor response to more traditional therapies (Table 2) (105, 106).

Canakinumab is a monoclonal antibody that specifically targets the IL-1β cytokine and a common treatment target in patients with sJIA. Patients with sJIA treated with canakinumab also remain at risk for MAS, suggesting that IL-1β is not the sole contributor to the pathogenesis of MAS (96). In comparison, IL-1α also signals via the IL-1 receptor (107). By blocking the receptor with anakinra, both IL-1α and IL-1β signals are dampened. While the importance of IL-1β in sJIA is widely accepted, IL-1α may be more important in stimulating the cytokine cascade in patients with MAS. Further research is needed to determine the efficacy of IL-1 blockade in treating MAS in non-sJIA patients.

Like many cytokines, the source(s) of IL-1 during MAS is unclear. Gene expression analysis of immune cells and murine tissues suggest that neutrophils may be better producers of IL-1β than monocytes, while an IL-1 family member, IL-18, may be largely produced by epithelial cells (108). Free IL-18 was shown to be highly elevated in the serum of MAS patients compared to patients with sJIA flare without MAS or familial HLH. In agreement with these findings, blockade of IL-18 receptor reduced inflammation in a murine model of MAS induced by repeated CpG injections (109). In addition, IL-18 inhibition with recombinant human IL-18 binding protein (IL-18BP) in combination with anakinra successfully improved life-threatening hyperinflammation in a patient with a dominant heterozygous mutation in NLRC4 (Table 2) (38). NLRC4 triggers the inflammasome, an innate immune complex that responds via caspase-1 activation and IL-1β and IL-18 secretion. Gain of function mutations, as seen in Familial Mediterranean Fever (FMF), result in hyperactivation of the NLRC4 inflammasone which can in turn result in MAS (102, 110). Adjunct therapy with mTOR inhibition (i.e., rapamycin) was reported in an infant with MAS refractory to anakinra and corticosteroids found to have an NLRC4 mutation (111).

Elevated free IL-18 may aid in the diagnosis of MAS, and as such, IL-18 blockade may be an effective cytokine-directed therapy in some forms of MAS. Of note, IL-18BP is not commercially available in the United States but has been used compassionately (38).

Hypercytokinemia correlates with a worse prognosis and is considered by many to be the main driver of disease pathology and subsequently the morbidity and mortality associated with MAS (112). Since macrophage activation appears to trigger the cytokine cascade in MAS (8), a solid understanding of the immunology and pathogenesis is critical to target-specific therapy. Known inducers of macrophage activation include toll-like receptor (TLR) ligands and cytokines (62, 77, 113) (Figure 1). The type of TLR stimuli and cytokines in the inflammatory milieu define the genetic programs, either pro- or anti-inflammatory adopted by macrophages in response to inflammatory stimuli (79).

Emerging studies in sJIA patients and in animal models of cytokine storm syndromes suggest TLR stimulation regulates cytokine activity via monocyte response. Gene expression analysis of Peripheral blood mononuclear cells (PBMC) from sJIA patients revealed an increased TLR/IL-1R signature and TLR2 expression (99, 114). Ablation of the TLR/IL-1R adaptor molecule Myd88 (115–117) reversed disease pathophysiology in models of fHLH (42, 118). Unlike fHLH, which typically presents in infancy due to one of many autosomal recessive gene mutations, MAS occurs across all ages and may present in the absence of a known pathogen or trigger. Two murine models were developed to better understand the role of TLR stimulation in MAS. Murine models show that repeated stimulation of TLR9 with CpG results in clinical MAS (41). In this model, monocytes were the main cells responsive to TLR9 stimulation which induced production of IL-12 (33). Further, IL-10 proved to be protective since blockade of IL-10R lead to fulminant MAS (Table 2) (33, 41). In a second model of MAS, TLR4 stimulation with LPS was shown to induce clinical symptoms consistent with MAS in IL-6 transgenic mice (119). These findings shed light on the combinatorial effect of TLR ligands and cytokines in the induction of pathogenesis in MAS.

## Dendritic Cells in MAS

The role of dendritic cells (DC) in disease pathogenesis is largely mediated by the ability of these cells to present antigen to T cells (120). Most of our knowledge of the role of DC in MAS originates from studies in murine models of fHLH. Similar to patients with fHLH, impaired NK cell degranulation resulting from mutations in *PRF1, UNC13D, STXBP2*, and *RAB27A* has been reported in patients with MAS (45, 48, 121–123).

The current view on the contribution of DC to disease pathogenesis HLH, arises from studies in perforin-deficient (*Prf*^−/^^−^) mice. Symptoms of fHLH can be reproduced by the infection of *Prf*^−/^^−^ mice with LCMV, resulting in a fatal hyperinflammatory response characterized by hyperproliferation of IFNγ-producing CD8^+^ T cells, which are central to disease pathogenesis (32, 34). Since T cell proliferation requires antigen presentation by DC, investigative studies focus on the mechanisms by which perforin regulates DC function.

Hermans et al. demonstrated that cytolytic T lymphocytes (CTL) regulate DC function by eliminating antigen-loaded DC and preventing their access to the lymph nodes, therefore acting as gate-keepers (124). Yang et al. later showed that this elimination was dependent on perforin, since in *Prf*^−/^^−^ mice activated CTL failed to eliminate antigen-loaded DC (125). This suggests that in LCMV-infected *Prf*^−/^^−^ mice, the extensive proliferation of CD8 T cells can result from continuous activation by antigen-presenting DC that cannot be eliminated by defective CTL. This hypothesis was further supported by Terrell et al. who showed that the antigen-presenting capacity of DC is increased in LCMV-infected *Prf*^−/^^−^ mice, along with the numbers of DC containing viral antigen. Additionally, transfer of *Prf* ^+/+^ CD8 T cells to LCMV-infected *Prf*^−/^^−^ mice reduced IFNγ production by CD8 T cells suggesting that CTL limit T cell activation likely by eliminating virus-infected DC (126). Furthermore, Lykens et al. demonstrated that the increased activation of cytotoxic CD8 T cells was not due to an intrinsic defect of activation threshold, but rather an enhanced presentation of antigen by DC (127). In line with these findings, persistence of antigen was found to be correlated with disease pathogenesis. In IFNγ knockout BALB/c mice infected with MCM virus, the severity of HLH-like symptoms was reduced in mice administrated with the antiviral cidofovir, further supporting the notion that antigen persistence drives constant antigen-presentation by DC (128). Collectively, these studies strongly propose that DC mediate disease pathogenesis in hosts with cytotoxic dysfunction. In cases where there is an infectious trigger, such as a viral infection, cytotoxic CTL fail to clear virus-infected DC. This leads to constant DC activation and antigen-presentation of viral antigens to T cells (31), which in turn respond by hyperproliferation and production of pro-inflammatory cytokines responsible for multi-organ failure seen in MAS.

## Conclusion

MAS is a potentially fatal inflammatory condition that can lead to multiorgan failure if inadequately treated. In the absence of validated diagnostic criteria, recognition is often delayed. A firm understanding of the pathogenesis of MAS can guide diagnosis and direct therapy toward target-specific treatment. A common hypothesis as to the pathophysiology of MAS proposes a defect in lymphocyte cytolytic activity. Normally, cytolytic cells induce cell apoptosis in abnormal cells. In the setting of an infection or inflammatory state, cytolytic cells may induce apoptosis in activated macrophages and T cells and serve to control the inflammatory response. A defect in cytolytic function may result in overstimulation of the immune system leading to the multi-organ failure seen in MAS.

The pro-inflammatory cytokine environment, particularly IL-6, has been shown to decrease NK cell cytolytic function. The inability of NK cells and cytolytic CD8 T cells to lyse infected and otherwise activated antigen presenting cells (APCs) results in prolonged cell-to-cell interactions and amplification of a pro-inflammatory cytokine cascade. The cytokine storm results in activation of macrophages, causing hemophagocytosis, as well as contributing to multi-organ dysfunction (Figure 1). Several cytokines, including TNF, IFNγ, and numerous interleukins (i.e., IL-1, IL-6, IL-18), have been implicated in the cytokine cascade. Specific heterozygous gene mutations in fHLH-associated cytolytic pathway genes (e.g., *PRF1, UNC13D*) have been linked to a substantial subset of MAS patients. These mutations cause defects in various proteins responsible for the production and transport of granules leading to apoptosis of target cells.

Historically, treatment of MAS focuses on controlling the underlying trigger, such as infection or sJIA treatment. However, not all cases present with a known pathogen or with a known etiology, making treatment of the underlying trigger impossible. It is important to understand the mechanism behind the uncontrolled cytokine storm seen in MAS to target specific cytokines upstream and prevent further stimulation of the activated macrophages. In addition to broadly immunosuppressive medications, such as corticosteroids and cyclosporine, cytokine specific therapy (e.g., IL-1 pathway blockade) may prove more effective in dampening the overly active immune system. Further studies and clinical trials are needed to better assess the role of pro-inflammatory cytokines in the pathogenesis of MAS and determine their clinical relevance.

## Author Contributions

CC: writing of the clinical and therapeutic sections, introduction and conclusion, organization of manuscript, primary editor; SA: writing of the basic science immunology and design of the figure; KN: writing of the basic science immunology, supervisor; RC: writing of the genetics sections, primary supervisor.

### Conflict of Interest Statement

RC is a co-Principal Investigator of an investigator initiated clinical trial of anakinra (funded by SOBI) to treat secondary HLH. The remaining authors declare that the research was conducted in the absence of any commercial or financial relationships that could be construed as a potential conflict of interest.
